# Supplementary material for: Sodium Leak Channel in Glutamatergic Neurons of the Lateral Parabrachial Nucleus Modulates Inflammatory Pain in Mice
Source: Int J Mol Sci. 2023 Jul 25;24(15):11907. doi: 10.3390/ijms241511907 (PMC10418977; doi:10.3390/ijms241511907)
Supplement: Supplementary file 1 [file ijms-24-11907-s001.zip › ijms-2512606-supplementary.pdf]

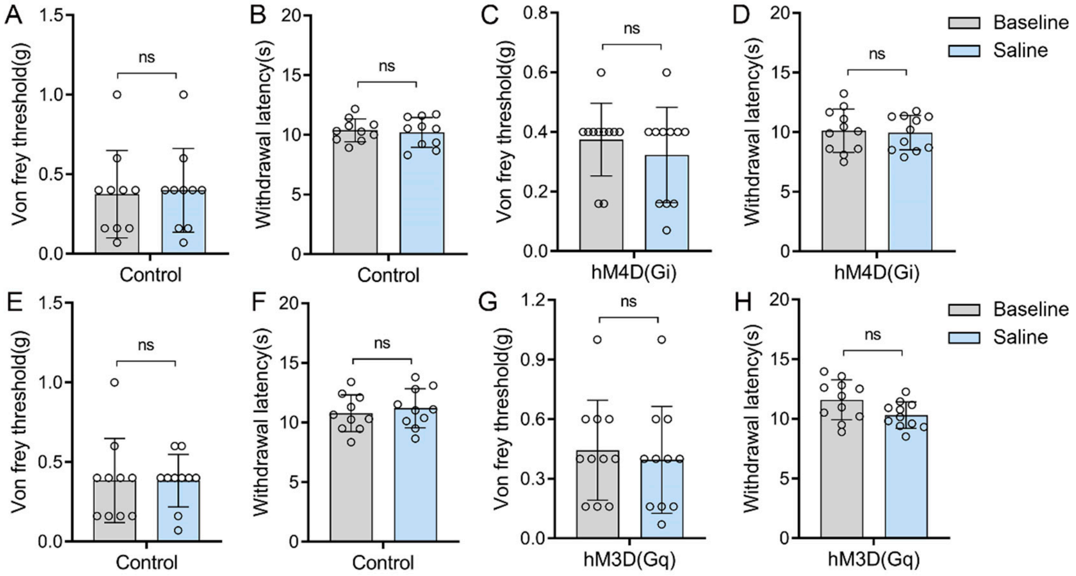

Figure S1. Saline injection did not affect pain thresholds in mice with chemogenetic manipulation of PBL glutaminergic neurons.
